# Soluble fms-like tyrosine kinase-1 and peripartum cardiomyopathy: an exploratory meta-analysis of association and diagnostic performance

**DOI:** 10.3389/fcvm.2026.1825532

**Published:** 2026-06-08

**Authors:** Triwedya Indra Dewi, William Kamarullah, Hawani Sasmaya Prameswari, Chaerul Achmad, Ruswana Anwar, Delvac Oceandy, Firman Fuad Wirakusumah

**Affiliations:** 1 Department of Cardiology and Vascular Medicine, Faculty of Medicine, Padjadjaran University, Hasan Sadikin General Hospital, Bandung, Indonesia; 2 Department of Obstetrics and Gynecology, Faculty of Medicine, Padjadjaran University, Hasan Sadikin General Hospital, Bandung, Indonesia; 3 Division of Cardiovascular Sciences, The University of Manchester, Manchester Academic Health Science Centre, Manchester, United Kingdom

**Keywords:** biomarker, meta-analysis, peripartum cardiomyopathy, sFlt-1, soluble fms-like tyrosine kinase-1

## Abstract

**Introduction:**

Peripartum cardiomyopathy (PPCM) is a rare cause of heart failure in late pregnancy and early postpartum, and biomarkers for risk stratification are lacking. Soluble fms-like tyrosine kinase-1 (sFlt-1), implicated in PPCM and pre-eclampsia, may be associated with PPCM and may have potential diagnostic relevance. This study aimed to evaluate the association between circulating sFlt-1 levels and PPCM and to provide an exploratory assessment of the diagnostic performance of sFlt-1 overall and according to pre-eclampsia status.

**Methods:**

We searched PubMed, Europe PMC, and ScienceDirect from inception to 15th February 2026 for studies reporting sFlt-1 in PPCM cases and pregnant or postpartum controls. Given the rarity of PPCM and the limited number of eligible studies, this review was designed as an exploratory meta-analysis. Random-effects models pooled odds ratios (ORs) for PPCM comparing high vs. low sFlt-1 and enabled subgroup analyses by pre-eclampsia. I Diagnostic performance was summarised using pooled sensitivity, specificity, and the area under the receiver operating characteristic curve (AUC).

**Results:**

Three studies (*n* = 226) met the criteria. High sFlt-1 levels were associated with increased PPCM risk [pooled OR 1.79; 95% confidence interval (CI) 1.22–2.64], although substantial heterogeneity was present across studies. In exploratory diagnostic analyses, performance was modest, with sensitivity 72% (95% CI 64%–80%), specificity 57% (95% CI 44%–68%), and AUC 0.71 (95% CI 0.67–0.75). In women with pre-eclampsia, the association with PPCM was stronger (OR 2.11; 95% CI 1.45–3.09) than in those without pre-eclampsia (OR 1.36; 95% CI 1.03–1.80), with higher sensitivity and specificity.

**Conclusion:**

Elevated sFlt-1 was associated with PPCM, with exploratory analyses suggesting somewhat better diagnostic discrimination in women with pre-eclampsia. However, because all included studies measured sFlt-1 in the postpartum period, these findings should not be interpreted as evidence for antepartum screening utility. Larger prospective studies with measurements during pregnancy and serial peripartum follow-up are needed before any clinical application can be considered.

**Systematic Review Registration:**

https://www.crd.york.ac.uk/PROSPERO/view/CRD42024535557, identifier (CRD42024535557).

## 1 Introduction

Peripartum cardiomyopathy (PPCM) is a rare but potentially life-threatening form of heart failure that occurs toward the end of pregnancy or in the months following delivery in women without another identifiable cause of cardiomyopathy (1, 2). Regardless of variable occurrence and fatality rates amid studies, PPCM constitutes one of the primary causes of pregnancy-related morbidity and mortality across borders (3–5). Several contributing factors encompassing age, multiparity, twin pregnancy, hypertension, and pre-eclampsia may affect the incidence, which differs by geographical region and ethnic background of the given study (6). Yet, the lack of particular biomarkers has further complicated the estimate of PPCM, which hindered us from anticipating this guise of malady.

Several recent investigations have found that PPCM is closely linked to endothelial dysfunction, which is caused by late-gestational hormone changes. Some experimental evidences also show a strong association between genetic predisposition, immunological response, and imbalanced oxidative stress during pregnancy, which activates multiple cardio-vasotoxic, inflammatory, and antiangiogenic factors that cause PPCM (7–9). Despite advances in comprehending the pathophysiological mechanisms of this entity, considerable fundamental gaps in knowledge still prevail.

Current evidence suggests that PPCM is linked to impaired vascular and myocardial adaptation during late pregnancy, involving angiogenic imbalance, endothelial dysfunction, oxidative stress, inflammation, and maternal susceptibility factors. Among the candidate mediators, soluble fms-like tyrosine kinase-1 (sFlt-1) has attracted particular interest because it is an antiangiogenic factor that binds vascular endothelial growth factor (VEGF) and placental growth factor (PlGF), thereby reducing proangiogenic signalling and promoting endothelial dysfunction (2, 10, 11).

This pathway is also central to pre-eclampsia, a condition that shares several clinical and biological features with PPCM and is a recognized risk factor for its development. These overlaps have raised interest in whether circulating sFlt-1 is associated with PPCM and whether it may provide useful discriminatory information, particularly in relation to pre-eclampsia status (11–13). However, whether sFlt-1 reflects a disease-specific signal for PPCM or a broader marker of pregnancy-related cardiovascular stress remains uncertain. Therefore, we performed an exploratory meta-analysis to examine the association between circulating sFlt-1 levels and PPCM and to provide a preliminary exploratory assessment of discriminatory performance, including according to pre-eclampsia status.

## 2 Materials and methods

### 2.1 Protocol and registration

This systematic review was conducted in accordance with the Cochrane Handbook for Systematic Reviews of Interventions and reported based on the Preferred Reporting Items for Systematic Reviews and Meta-Analysis (PRISMA) (14). The protocol was registered at the International Prospective Register of Systematic Reviews (PROSPERO), under identification number CRD42024535557.

### 2.2 Search strategy

**Figure 1:**
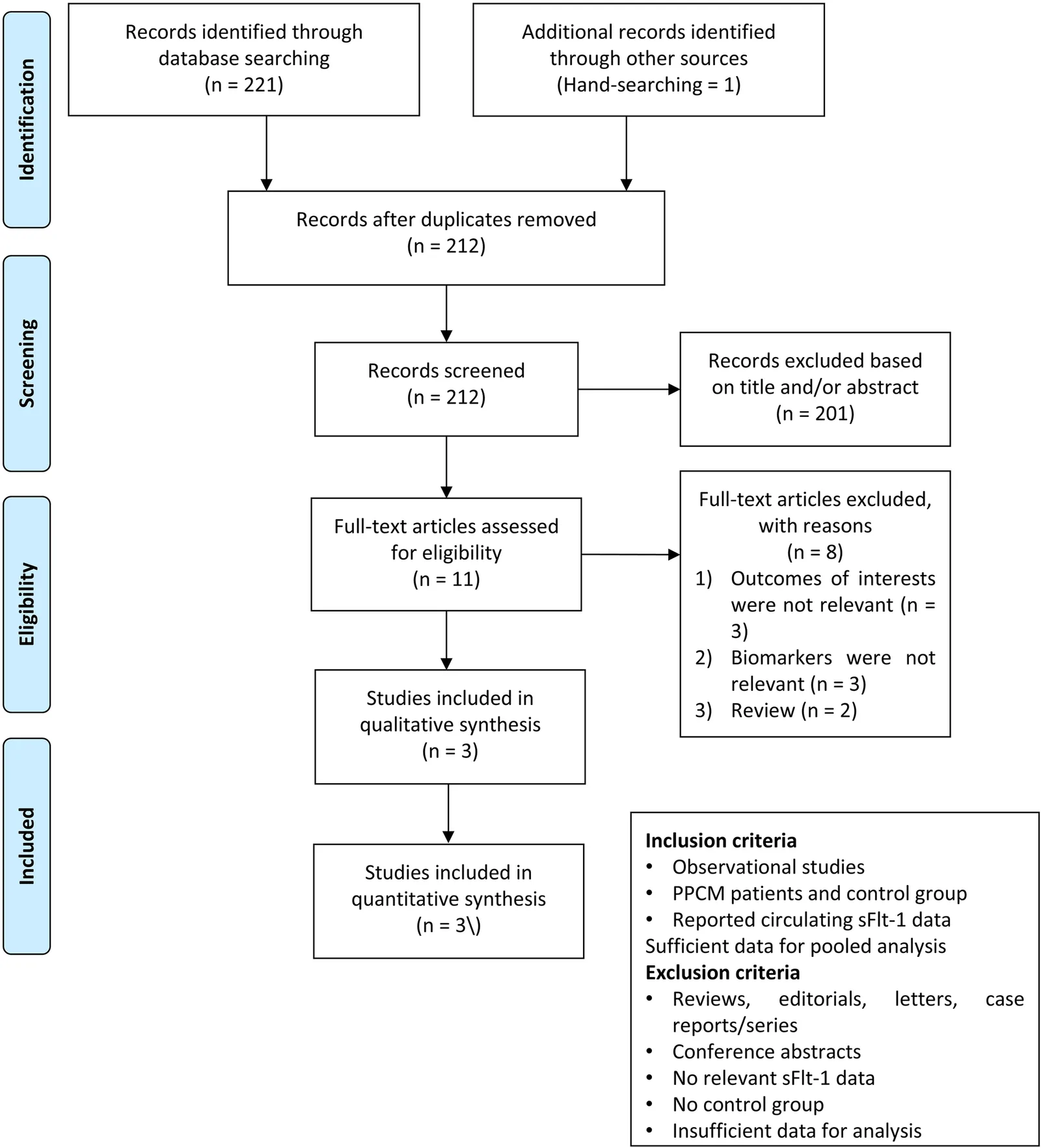
Flow chart of study selection.

Two independent investigators conducted a systematic search of PubMed, Europe PMC, and ScienceDirect from inception to February 15th 2026. The search query includes keywords and search phrases that involve (soluble fms-like tyrosine kinase-1 OR sFlt-1) AND (peripartum cardiomyopathy). We employed the least number of keywords possible to optimize the initial area of inquiry and ensure the greatest number of articles were recorded. To widen our search results, we also employed hand searches through the references of the included articles. The PRISMA criteria were implemented in our search, and Figure 1 depicts the search and screening processes.

### 2.3 Study selection

In this meta-analysis, we considered studies with particular information studying the sFlt-1 in predicting PPCM. The detailed inclusion criteria were as follows: (1) prospective or retrospective observational studies (case-control or cohort); (2) studies including patients with PPCM; (3) reported the key exposure between high and low sFlt-1, defined as an increment beyond cut-off point reported in each study in a comparative manner between the aforementioned variable. Our investigation required studies to publish risk estimation data with 95% confidence intervals (CI) or report sufficient data to compute the effect size. To perform a diagnostic-test accuracy meta-analysis, the study must provide apposite data on confusion matrix units to complete the statistical calculation related to the formula. Studies that did not provide enough of the aforementioned data were omitted from our analysis. Our meta-analysis also excluded review papers, editorials, comments, case reports/series, meta-analyses, and conference abstracts.

### 2.4 Data extraction and risk of bias assessment

We extracted the data from the eligible studies, including baseline characteristics of the included studies (first author's name, the country in which the study was conducted, study design, study participants and control population characteristics, PPCM diagnostic criteria, exclusion criteria, total participants, age, race, multiparity, twin pregnancies, gestational hypertension, pre-eclampsia, body mass index (BMI), assay platform used to measure sFlt-1 in blood). When available, we also extracted the study-specific definition of pre-eclampsia and the timing of sFlt-1 measurement relative to delivery and PPCM diagnosis.

The Newcastle-Ottawa Scale (NOS) was implemented by the authors to independently assess the possibility of bias in each study. A study with a total score of seven or above was deemed bias-free. Research with a total score of six or less was considered to be biased and thus excluded from the research. Author discussion was utilized to settle quality rating disagreements (15).

### 2.5 Statistical analysis

For statistical analysis, STATA 17.0 (Software for Statistics and Data Science) was used. The effect estimates in this meta-analysis were expressed as odds ratios (ORs) with 95% CI. The random-effects models were used to pool the overall effect magnitude owing to the fact that the inter-study heterogeneity was high and small included number of studies. Mean differences were translated into effect sizes using the Cohen method and the random-effects inverse-variance model with DerSimonian-Laird estimate. The I^2^ index was used to examine inter-study heterogeneity, and an I^2^ statistic of more than 50% or a *P*-value less than 0.10 disclosed significant heterogeneity (16).

We additionally conducted a subgroup meta-analysis according to the presence or absence of pre-eclampsia to explore one clinically relevant source of heterogeneity. Meta-regression was not performed because only three studies were eligible for inclusion; with fewer than 10 studies, meta-regression is generally considered unreliable, underpowered, and susceptible to unstable estimate (16). For exploratory diagnostic-test accuracy analysis, 2 × 2 contingency-table data were extracted or reconstructed from each study when possible. Study-level sensitivity and specificity were calculated and presented in forest plots. A summary receiver operating characteristic (SROC) curve was then generated to provide an overall descriptive summary of diagnostic discrimination, and the area under the curve (AUC) was reported. Because of the limited number of eligible studies, differences in assay platforms, cut-off values, and timing of blood sampling, these diagnostic analyses were considered exploratory and were interpreted cautiously rather than as evidence of established clinical utility. Furthermore, the Egger test was utilized to quantify the publication bias. All statistical analyses were two-sided, with statistical significance attained by a *P*-value <0.05. Given the limited number of eligible studies and the variability in study design, assay platforms, cut-off values, and timing of blood sampling, the diagnostic-test accuracy analysis was considered exploratory and was interpreted cautiously.

## 3 Results

### 3.1 Study selection and baseline characteristics

The primary search results yielded 222 articles. After screening the titles and abstracts, 11 papers were identified for further assessment based on the promulgated inclusion criteria. Ultimately, 3 studies were included in our analysis (17–19). Figure 1 depicts the results of the literature search, including the basis for excluding certain publications. The baseline characteristics of the included studies in the final review were presented in Table 1. In general, the PPCM definition applied across studies was identified as having symptoms of heart failure in the last months of pregnancy or the first 5 months post-partum, with no other established cause of heart failure, in addition to a left ventricular ejection fraction of ≤45% on echocardiography (18, 19). The majority of studies used a prospective study design, with the mean age of participants in the case group was insentiently similar (31.6 ± 3.5 years old) to the control group (32.2 ± 2.7 years old). Most of the participants were African ancestry (76%), one of the studies excluded pre-eclampsia from its investigation (19). Importantly, all included studies measured sFlt-1 during the postpartum period rather than during pregnancy. Diverse sFlt-1 assay platforms were also acknowledged across the included research.

**Table 1:** Baseline characteristics of the included studies (PPCM cases vs controls).

No.	Author (year)	Country	Study design	Study population characteristics	Control population characteristics	PPCM diagnostic criteria	Definition of pre-eclampsia	Exclusion criteria	Total participants	Age (years)	Race	Multiparity (%)	Twin pregnancies (%)	Gestational hypertension (%)	Pre-eclampsia (%)	BMI (kg/m^2^)	Assay platform	Timing of sFlt-1 measurement relative to delivery/PPCM diagnosis	NOS
1	Ersbøll et al. (2021) (17)	Denmark	Retrospective observational study	Singleton pregnancy with PPCM	Healthy, uncomplicated pregnant women with singleton fetus	Defined according to prior PPCM diagnosis in medical records; detailed criteria not explicitly reported in the publication	Not explicitly reported	Not explicitly reported	56 (28 vs 28)	30.7 ± 6 vs 31 ± 5.2	African: 100% vs 96%	18% vs 21%	NR	7% vs 0%	39% vs 0%	28.3 ± 6.4 vs 21.3 ± 1.8	V-PLEX Custom Human Biomarkers Assay (Meso Scale Discovery®)	Case & Control: Post-partum (91 months)	8
2	Mebazaa et al. (2017) (19)	South Africa	Prospective observational study	Singleton pregnancy with PPCM	Healthy, uncomplicated pregnant women with singleton fetus	(1) Symptoms of congestive heart failure that developed in the last months of pregnancy or during the first 5 months post-partum; (2) no other identifiable cause for heart failure; (3) left ventricle ejection fraction ≤45% by transthoracic echocardiography; and (4) sinus rhythm	Excluded patients with postpartum cardiac failure associated with hypertension, including pre-eclampsia	(1) Significant organic valvular heart disease; (2) systolic blood pressure >160 mmHg or diastolic blood pressure >100 mmHg; (3) clinical conditions other than cardiomyopathy that could increase plasma concentrations of inflammatory markers; (4) severe anemia (hemoglobin <9 g/dL); (5) patients with post-partum cardiac failure associated with any form of hypertension, including pre-eclampsia	113 (83 vs 30)	28.7 ± 7.5 vs 30.3 ± 10.1	African: 100%	NR	NR	0%	0%	NR	Roche® Cobas E601 Analyzer	Case: within 4 h of the unscheduled admission for acute dyspnea (median: 4 weeks post-partum); Control: within 24 h after delivery	8
3	Goland et al. (2016) (18)	Israel	Prospective observational study	Singleton pregnancy with PPCM	Healthy, uncomplicated pregnant women with singleton fetus	The development of idiopathic cardiomyopathy during pregnancy or within 5 months of delivery with LV systolic dysfunction as assessed by echocardiography with LVEF <45%	Not explicitly reported	NR	57 (28 vs 29)	35.5 ± 6 vs 35.2 ± 8.1	European: 59% vs 62%; African: 28% vs 31%; Ethiopian: 14% vs 7%	NR	24% vs 14%	21% vs 17%	17% vs 7%	NR	R&D® Systems	Case & Control: Post-partum (12 months)	8

BMI, body mass index; kg/m^2^, kilograms divided by the square of the height in meters; NOS, Newcastle-Ottawa Scale; NR, not reported; PPCM, peripartum cardiomyopathy.

### 3.2 Soluble fms-like tyrosine kinase-1 and peripartum cardiomyopathy

**Figure 2:**
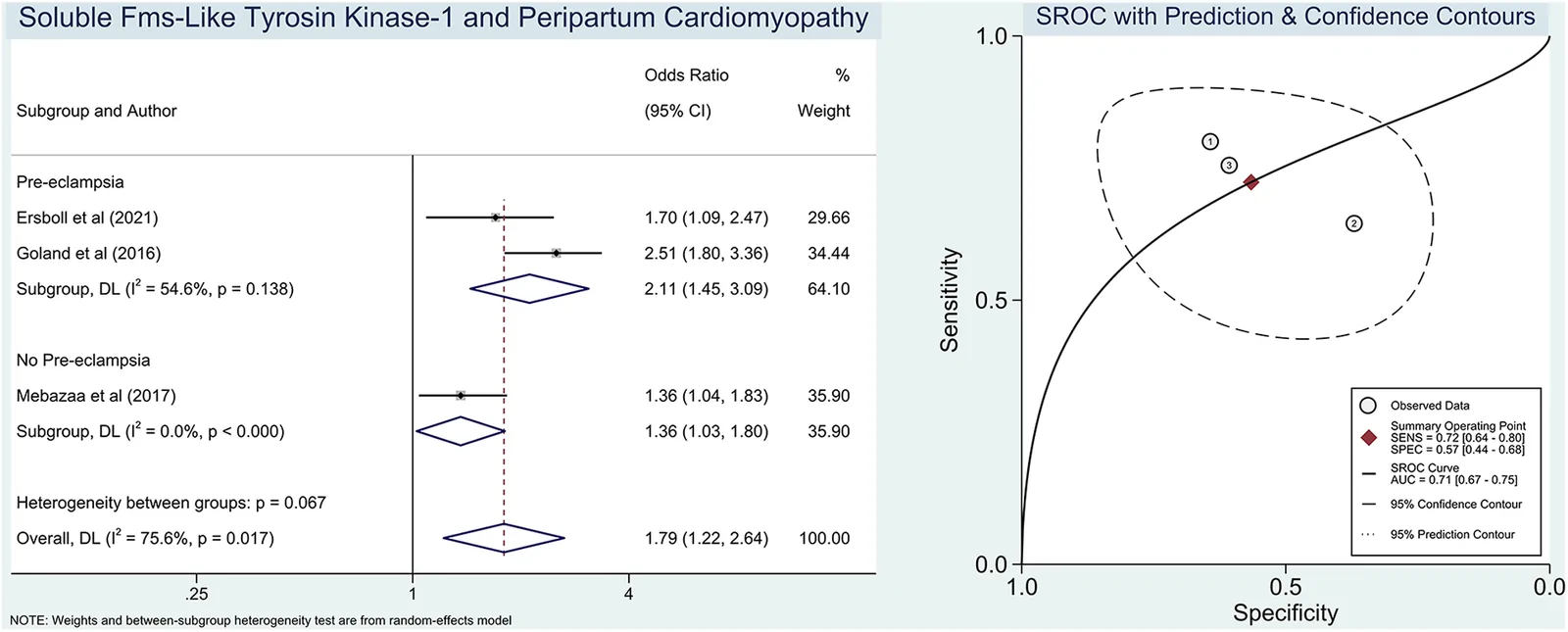
Forest plot of merged odds ratios and summary of receiver operating characteristic (SROC) curve for soluble fms-like tyrosine kinase-1 (sFlt-1) in predicting peripartum cardiomyopathy.

A total of three prospective and retrospective observational studies evaluated the correlation between sFlt-1 and PPCM. Our merged analysis suggested that population with high sFlt-1 had a significant increased risk of PPCM (OR = 1.79 (95% CI = 1.22–2.64); *P* = 0.003), although heterogeneity was substantial (I^2^ = 75.6%; P for heterogeneity=0.017), indicating important between-study variability. This heterogeneity was likely influenced by differences in assay platforms, study-specific cut-off values, study populations, and timing of postpartum blood sampling. Furthermore, diagnostic-test accuracy meta-analysis results showed that high sFlt-1 had a sensitivity of 72% (95% CI, 64%–80%), specificity of 57% (95% CI, 44%–68%), and AUC of 0.71 (95% CI, 0.67–0.75) in correlation with PPCM (Figure 2).

Pooled research based on the concurrent coexistence of pre-eclampsia revealed that the pooled OR in the prior mentioned subgroup was greater and statistically significant (OR = 2.11 (95% CI = 1.45–3.09); *P* < 0.001; I^2^ = 54.6%, P-heterogeneity=0.138) than the pooled effect size in the non-pre-eclampsia subgroup (OR = 1.36 (95% CI = 1.03–1.80); *P* = 0.033; I^2^ = 0%, P-heterogeneity < 0.001) (Figure 2). Exploratory subgroup analyses suggested somewhat better diagnostic performance of sFlt-1 in studies including women with pre-eclampsia than in the study excluding pre-eclampsia, as evidenced by raised further sensitivity and specificity compared to the non-pre-eclampsia subgroup (Table 2). However, this analysis should be interpreted with considerable caution because the pre-eclampsia subgroup was based on only two studies and the non-pre-eclampsia subgroup on a single study, resulting in limited statistical power and restricted inferential value.

**Table 2:** Summary results of soluble fms-like tyrosine kinase-1 (sFlt-1) in predicting peripartum cardiomyopathy.

Author (year)	Odds ratio (95% CI)	Sensitivity (95% CI)	Specificity (95% CI)
Pre-eclampsia subgroup			
**Ersbøll et al. (2021)** (17)	1.70 (1.09–2.47) (*P* < 0.001)	80% (65%–90%)	64% (44%–81%)
**Goland et al. (2016)** (18)	2.51 (1.80–3.36) (*P* < 0.001)	76% (61%–87%)	61% (41%–78%)
No pre-eclampsia subgroup			
**Mebazaa et al. (2017)** (19)	1.36 (1.03–1.80) (*P* = 0.033)	65% (45%–81%)	61% (41%–78%)

CI, confidence interval.

.

### 3.3 Publication bias

We were unable to conduct a qualitative analysis of funnel plots to identify publication bias due to a paucity of research in our analysis. Hence, the Egger test was used to quantitatively detect publication bias, and our findings revealed that a small study effect was not identified.

## 4 Discussion

To the best of our knowledge, no previous meta-analysis has specifically synthesized the available evidence on circulating sFlt-1 in PPCM, including analyses according to pre-eclampsia status. In our analysis of women with PPCM, we found that plasma levels of sFlt-1 were significantly higher compared with age-matched control groups of women with uncomplicated pregnancies [125.6 ± 45.5 vs. 93.6 ± 51.2 pg/mL (picograms per millilitre)]. The data analysis revealed that greater levels of sFlt-1 increased the probability of developing PPCM. On the basis of the above measures, we also observed that higher sFlt-1 levels appeared to show greater discriminatory performance in patients with concurrent pre-eclampsia. While PPCM contributes considerably to maternal and perinatal morbidity and death, it is, therefore, essential to identify women who are at risk of developing PPCM to minimize unfavourable pregnancy outcomes through early intervention. To date, reliable and disease-relevant biomarkers for identifying women at risk of PPCM remain limited (10, 20). It is still precarious whether sFlt-1 is sufficiently sensitive or specific to be used for diagnosing PPCM, nor the aforementioned biomarker can distinguish between PPCM with and without pre-eclampsia (17–19), as will be captivatingly discussed in the remainder of this article.

Beyond PPCM specifically, our findings may also be interpreted within the broader framework of maternal cardiovascular adaptation to pregnancy. Pregnancy is increasingly recognized as a physiological cardiovascular stress test, during which the maternal circulation must accommodate marked changes in plasma volume, cardiac output, vascular tone, and endothelial function. In this setting, sFlt-1 may represent not only a marker of placental antiangiogenic signalling, but also a broader marker of maternal cardiovascular and endothelial stress. This interpretation is supported by studies showing that angiogenic imbalance during pregnancy, particularly elevated sFlt-1 in hypertensive disorders such as pre-eclampsia, is associated with adverse maternal cardiovascular remodelling and increased long-term cardiovascular risk after pregnancy. Therefore, the observed association between sFlt-1 and PPCM may reflect a more general antiangiogenic and cardiovascular stress phenotype, of which PPCM may represent one severe manifestation in susceptible women (21–23).

These mechanisms may not be unique to PPCM, but may instead form part of a wider spectrum of pregnancy-related cardiovascular maladaptation in which angiogenic imbalance contributes to endothelial dysfunction, myocardial stress, and reduced maternal cardiovascular reserve. Oxidative stress-mediated cleavage of the hormone prolactin into a smaller physiologically active fragment, 16-kDa (kilodalton) prolactin, has primed as the major factor initiating PPCM. This “rule-the-roost” vascular-hormonal hypothesis proposes that 16-kDa prolactin will upregulate miR-146a (micro ribonucleic acid (RNA)-146a), leading to several toxic effects on the endothelium, coupled with sFlt-1, which will reduce various proangiogenic factors, resulting in diminished vascular and cardiac function and subsequent heart failure in late pregnancy and post-partum (10, 24, 25).

Despite its positive findings and plausible explanations between these two prior mentioned variables, there are a few key points to consider before drawing any conclusions from our study. Given the evidence that there is a substantial epidemiological link between pre-eclampsia and PPCM, subgroup analysis for this possible confounder was also undertaken to assure the true direct effect regarding the aforementioned outcome (26, 27). Our findings corroborate this evidence, indicating that the pooled effect size for the relationship between sFlt-1 along with PPCM was higher in the pre-eclampsia subgroup than in the non-pre-eclampsia subgroup. In terms of diagnostic accuracy, it was further established that sFlt-1 persevered better when pre-eclampsia was present, as presented by a greater value of sensitivity and specificity compared to the non-pre-eclampsia group.

Several conceivable rationales or else viable explanations might be raised upon this matter. To begin, the non-pre-eclampsia subgroup is represented by a study conducted by Mebazaa et al. (19), who collected blood samples for analysis shortly after the diagnosis of PPCM was obtained (median: 4 weeks post-partum) and compared them to blood results collected from women with uncomplicated pregnancies within 24 h of delivery. It should be emphasized that sFlt-1 levels are strongly impacted by particularly specific physiological changes that occur throughout the peripartum period. For instance, the concentration of sFlt-1 is dramatically elevated throughout the end of pregnancy and in the early days following conception but returns to its baseline (non-pregnant) level within 5 to 6 weeks post-partum (28, 29). Thus, the low sensitivity and specificity found in this subgroup may be related, at least in part, to similarities in the time of blood sampling between the post-partum controls and the PPCM patients, in which sampling was undertaken during the heightened physiological rise period.

A particularly important limitation is that all included studies measured sFlt-1 after delivery rather than during pregnancy before PPCM onset. This substantially restricts any inference regarding antepartum screening. A screening biomarker must be measured at a clinically actionable time point before disease recognition, whereas postpartum sampling captures biomarker levels after delivery, after placental separation, and often after or near the time of PPCM diagnosis. Because sFlt-1 is strongly influenced by placental biology and changes rapidly across the peripartum and postpartum periods, postpartum concentrations may reflect physiological clearance, residual placental signalling, hemodynamic stress, or the consequences of established disease rather than a preclinical state that could enable screening. Therefore, an observed postpartum association between elevated sFlt-1 and PPCM does not establish that antepartum measurement would identify women at risk before clinical presentation (28, 29).

The generally higher diagnostic value of sFlt-1 in the serum in predicting the PPCM in the pre-eclampsia subgroup could also be explained by the later timing of blood sampling (≥12 months post-partum) in comparison to control groups in relation to delivery, which may simply reflect the normal clearance of sFlt-1 post-partum (17, 18). Nonetheless, our findings may suggest that the diagnostic value of sFlt-1 is hypothesized to be affected in a time-dependent fashion, given that the spectrum of values between both subgroups differs significantly. This yet unveils an up in the air evidence gap corresponding to that sFlt-1 is still categorized as a novel biomarker in the domain of PPCM, leaving some unanswered question concerning “how early is early” to run the test. It is either too early an examination that demonstrates scarce diagnostic value in the non-pre-eclampsia subgroup, or perhaps PPCM without pre-eclampsia shares a distinct underlying pathophysiological mechanism within.

There is debate concerning involving patients with pregnancy-associated hypertension (e.g., pre-eclampsia) in PPCM cases, as previous clinical studies coupling pre-eclampsia to the development of PPCM have rarely been separately compared and contrasted, although the longitudinal clinical and functional outcomes of PPCM associated with pre-eclampsia and PPCM that is not associated with pre-eclampsia are diverge (7, 27). Our findings are consistent with the concept that PPCM associated with pre-eclampsia may be understood within a two-hit framework, in which systemic antiangiogenic signalling during late pregnancy interacts with maternal susceptibility, including impaired local proangiogenic defences in the heart (2, 30). We further argue that higher sFlt-1 levels provide a challenge for the myocardium in all pregnancies, explaining why the peripartum period and other factors put women at risk of developing PPCM, even in the absence of pre-eclampsia Prior literature has suggested that conditions associated with greater antiangiogenic burden, such as twin pregnancies, as well as repeated pregnancy exposures and inflammatory states, may contribute to PPCM susceptibility (31). Nevertheless, these factors were not directly evaluated in the present meta-analysis and are mentioned here as mechanistically plausible considerations rather than findings derived from our pooled analysis. This also demonstrates that, while blood samples were collected after the placenta was removed post-delivery, sFlt-1 is thought to be generated largely by the placenta, yet it is also possible that it is produced by placental remains and pericytes from other tissues (32, 33). However, these findings should be interpreted cautiously. The evidence base was limited to three small observational studies, and the pooled estimates of diagnostic performance are best viewed as preliminary and hypothesis-generating rather than definitive evidence of clinical utility.

### 4.1 Clinical implications

In this exploratory meta-analysis, higher sFlt-1 levels were associated with increased odds of PPCM. However, association alone does not establish clinical diagnostic usefulness. The pooled diagnostic estimates observed in this review, sensitivity 72%, specificity 57%, and AUC 0.71, reflect only modest discrimination and are insufficient to support clinical implementation of sFlt-1 as a standalone diagnostic or screening biomarker for PPCM at present. Although sensitivity appeared somewhat higher in the presence of pre-eclampsia, specificity remained limited, which restricts rule-in value and raises concern regarding false-positive classification. Accordingly, sFlt-1 should currently be regarded as an investigational biomarker rather than a test ready for routine clinical application (34). In the presence of pre-eclampsia, sFlt-1's diagnostic value arose in terms of sensitivity (ranging from 76 to 80%), but low specificity in both subgroups. Thus, it is best to rule out rather than rule in the possibility of the outcome. The fact that approximately 20% of women with PPCM have co-incident pre-eclampsia (35), our findings add to the body of knowledge that might assist clinicians in predicting the development of PPCM and counselling patients more accurately.

### 4.2 Limitations

Several limitations warrant emphasis. First and most importantly, only three studies met the eligibility criteria, yielding a total sample size of 226 participants. This is a severe limitation that markedly reduces statistical power, widens uncertainty around pooled estimates, and limits the generalizability of the findings across populations and clinical settings. Accordingly, the present meta-analysis should be interpreted as exploratory and hypothesis-generating rather than confirmatory. Nonetheless, we believe this synthesis remains valuable because it provides a structured summary of a small and fragmented literature, highlights the current lack of robust evidence, and identifies methodological issues that should be addressed in future studies. Second, all included studies measured sFlt-1 in the postpartum period, which is a major limitation for any interpretation related to screening or risk stratification during pregnancy. Because sFlt-1 is predominantly placenta-derived and undergoes substantial peripartum and postpartum changes after delivery, postpartum values cannot be assumed to represent antepartum biomarker status. Moreover, postpartum measurements were obtained after placental separation and often near or after PPCM diagnosis, making it impossible to determine whether elevated sFlt-1 preceded disease onset or merely accompanied established disease or postpartum physiological change. For this reason, our findings should not be extrapolated to antepartum screening utility. Third, substantial heterogeneity was present and was likely attributable to differences in assay platforms, cut-off definitions, study populations, and timing of blood sampling in relation to delivery and PPCM diagnosis. Fourth, the diagnostic performance analysis was based on sparse data and should therefore be interpreted as exploratory. Moreover, the pooled diagnostic estimates were only modest, indicating that sFlt-1 is not yet suitable for clinical use as a standalone diagnostic or screening biomarker for PPCM. Fifth, most participants were of African ancestry, which may limit generalizability to other populations. Future prospective studies should include serial antepartum and postpartum measurements, standardized sampling protocols, and clinically relevant thresholds to determine whether sFlt-1 has value for risk stratification before clinical presentation.

## 5 Conclusions

In conclusion, elevated sFlt-1 levels were associated with PPCM. Although exploratory subgroup analyses showed numerically larger association estimates and discriminatory indices in studies including women with pre-eclampsia, these observations were based on very small subgroups and are susceptible to residual confounding. Therefore, they should be regarded as descriptive and hypothesis-generating only, rather than as evidence of superior performance, causality, or predictive value. Larger prospective studies with standardized sampling during pregnancy and across the peripartum period are needed to clarify the clinical relevance of sFlt-1 in PPCM.

## Data Availability

The data analyzed in this study were derived from published articles cited in the reference list. Extracted data supporting the findings of this study are included in the article. Further inquiries can be directed to the corresponding author.
