# Correction: Soluble fms-like tyrosine kinase-1 and peripartum cardiomyopathy: an exploratory meta-analysis of association and diagnostic performance

**DOI:** 10.3389/fcvm.2026.1944285

**Published:** 2026-08-10

**Authors:** Triwedya Indra Dewi, William Kamarullah, Hawani Sasmaya Prameswari, Chaerul Achmad, Ruswana Anwar, Delvac Oceandy, Firman Fuad Wirakusumah

**Affiliations:** 1 Department of Cardiology and Vascular Medicine, Faculty of Medicine, Padjadjaran University, Hasan Sadikin General Hospital, Bandung, Indonesia; 2 Department of Obstetrics and Gynecology, Faculty of Medicine, Padjadjaran University, Hasan Sadikin General Hospital, Bandung, Indonesia; 3 Division of Cardiovascular Sciences, The University of Manchester, Manchester Academic Health Science Centre, Manchester, United Kingdom

**Keywords:** biomarker, meta-analysis, peripartum cardiomyopathy, sFlt-1, soluble fms-like tyrosine kinase-1

In the published article, there was a mistake in the Funding statement. The funding statement was displayed as “The author(s) declared that financial support was not received for this work and/or its publication.”. The correct statement is “The author(s) declared that financial support was received for this work and/or its publication. This publication charge is funded by Unpad through the Indonesian Endowment Fund for Education (LPDP) on behalf of the Indonesian Ministry of Higher Education, Science, and Technology. This was managed under the EQUITY Program (Contract No. 4303/B3/DT.03.08/2025 and 3927/UN 6.RKT/HK.07.00/2025”

The original version of this article has been updated.

